# Association of genetic polymorphisms with acute kidney injury after cardiac surgery in a Southeast Asian population

**DOI:** 10.1371/journal.pone.0213997

**Published:** 2019-04-11

**Authors:** Kah Ming Eddy Saw, Rui Ge Roderica Ng, Siew Pang Chan, Yi Hui Ang, Lian Kah Ti, Tsong Huey Sophia Chew

**Affiliations:** 1 Department of Anaesthesiology, Singapore General Hospital, Academia, Level 5, Singapore; 2 Cardiovascular Research Institute, National University Health System, Singapore; 3 Department of Mathematics and Statistics, College of Science, Health and Engineering, La Trobe University, Melbourne, Victoria, Australia; 4 Department of Anaesthesia, National University Health System, Singapore; 5 Yong Loo Lin School of Medicine, National University of Singapore, Singapore; 6 Duke-NUS Medical School, Singapore; Taipei Medical University Hospital, TAIWAN

## Abstract

**Introduction:**

Genetic polymorphisms are important in explaining the wide interpatient variability that exists in the development of acute kidney injury (AKI) post cardiac surgery. We hypothesised that polymorphisms in 4 candidate genes, namely angiotensin-converting enzyme (ACE), apolipoprotein-E (ApoE), interleukin-6 (IL-6), and tumour necrosis factor-alpha (TNF-α) are associated with AKI.

**Methods:**

870 patients who underwent cardiac surgery in Singapore were analysed. All patients who fulfilled stage 1 KDIGO criteria and above were considered to have AKI. This was investigated against various demographic, clinical and genetic factors.

**Results:**

Increased age, history of hypertension, anaemia and renal impairment remained important preoperative risk factors for AKI. Intraoperatively, longer cardiopulmonary bypass (CPB) time and the use of intra-aortic balloon pump (IABP) were shown to be associated with AKI. Among the genetic factors, ACE-D allele was associated with an increased risk of AKI while IL6-572C allele was associated with a decreased risk of AKI.

**Conclusion:**

ACE-D allele was associated with the development of AKI similar to other studies. On the other hand, IL6-572C was shown to have a protective role against the development of AKI, contradictory to studies done in the Caucasian population. This contradictory effect of IL6-572C is a result of a complex interplay between the gene and population specific modulating factors. Our findings further underscored the necessity of taking into account population specific differences when developing prediction models for AKI.

## Introduction

Acute kidney injury (AKI) is a common complication after cardiac surgery, occurring in up to 17 to 49% of patients, as depicted in previous studies. It is associated with significant morbidity and mortality, increased length of hospitalisation and imposes a significant healthcare burden. Furthermore, 2 to 6% of patients require dialysis after developing AKI post cardiac surgery, with a significant number progressing to end stage renal disease [1–7].

The mechanisms involved in the development of AKI after cardiac surgeries are complex. They principally revolve around the interplay between acute and chronic clinical factors [1–7]. Acute insults sustained by the kidney include those related to cardiopulmonary bypass (CPB) and atheroembolism, both of which results in ischemic and/or inflammation mediated renal injury. Chronic risk factors such as diabetes, hypertension, and pre-existing renal impairment heighten the risk posed by these acute insults.

With AKI posing a significant healthcare burden, several authors have developed risk stratification models for AKI. The goals of such models have been to identify the “at risk” patient for developing AKI. It is believed that early intervention can reduce the severity of kidney injury among such “at risk” patients [8,9].

However the current models are based on clinical factors which only explain a small part of interpatient variability and susceptibility. Stafford-Smith et al found that a combined clinical and genetic model was able to explain up to 20% of the variability in the development of AKI [10]. The genetic polymorphisms that were identified included angiotensin-converting enzyme (ACE), interleukin-6 (IL-6), apolipoprotein-E (ApoE), and tumour necrosis factor-alpha (TNF-α). Further studies have since been completed, supporting the role genetic polymorphisms play in the pathogenesis of AKI [11–14].

Currently there are no prediction models that cater to our Southeast Asian population. With a view of developing our own population specific model in the future, we investigated whether polymorphisms in ACE, IL-6, ApoE and TNF-α genes had similar effects on AKI after cardiac surgery.

## Methods

### Study population

With SingHealth Centralised Institutional Review Board (CIRB) [IRB number 2008/137/D] approval and written informed consent from the patients, we prospectively followed up 1000 patients who had undergone cardiac surgery (coronary artery bypass grafting (CABG), valve surgery or combined CABG and valve surgery) between August 2008 and July 2010 at the two main tertiary heart centres in Singapore.

Patients who were already on either haemodialysis or peritoneal dialysis were excluded. Patients who were not of Chinese, Malay or Indian ethnicity were also excluded from analysis.

### Data collection

All perioperative data were prospectively collected and collated in a secure central database. Full time personnel who were blinded to the study details analysed the outcomes.

### Renal function assessment and perioperative renal data

The primary outcome was post-operative AKI as defined by Kidney Disease: Improving Global Outcomes (KDIGO) [15]. Pre-operative serum creatinine level was obtained within 7 days of surgery or repeated within 24 hours prior to surgery if there were material changes in the patient’s condition. The peak post-operative serum creatinine was obtained within 7 days after surgery. All patients who fulfill KDIGO stage 1 and above (ie, had at least a 50% increase from the pre-operative baseline serum creatinine level to the peak post-operative serum creatinine level), were considered to have AKI.

### Anaesthesia and surgical management

Perioperative surgical management and clinical practices at both institutions were similar and followed international practices. Typically, anaesthesia was induced with intravenous induction agents (etomidate or propofol) and maintained with a balanced anaesthetic regime of low-dose fentanyl (10–20μg/kg) and volatile agents (primarily sevoflurane). Conventional CPB circuits with roller pumps, membrane oxygenators, heat exchangers, venous reservoirs, cardiotomy suction and arterial blood filters were used. The volumes of priming solution used in the CPB circuits were typically between 1300 and 1400mL. Cardiac bypass perfusion targets were mild-to-moderate hypothermia (32–35°C), haematocrit levels of ≥22%, activated clotting times of >400 seconds, glucose levels of <10mmol/L, non-pulsatile flow rate of 2.2–2.4L/min/m^2^, and mean arterial pressure of 50-70mmHg. Myocardial protection was achieved with cold blood cardioplegia. Aprotinin was not used in any of the patients.

### Candidate polymorphism selection and isolation of genomic DNA

Genetic polymorphisms in 4 candidate genes which had been shown to be associated with AKI–namely angiotensin-converting enzyme (ACE), apolipoprotein E (ApoE), interleukin-6 (IL-6) and tumour necrosis factor-alpha (TNF-α)–were analysed in 2 batches (June to July 2011 for the first batch while the second batch was done from July to October 2013).

Blood samples were collected preoperatively and genomic DNA was isolated using the Maxwell 16 MDx Research System, namely the Maxwell 16 MDx Instrument and the Maxwell 16 Blood DNA Purification Kit (Promega, WI, USA).

Polymerase chain reaction (PCR) was used to detect the 2 ACE gene alleles, insertion (I) and deletion (D), corresponding to 490 and 190bp PCR products respectively. PCR products were then separated using gel electrophoresis.

The PCR restriction fragment length polymorphism (RFLP) method was used to identify the ε2, ε3, and ε4 genotypes of ApoE gene. Restriction enzyme Hhal was used to digest the PCR products and the RFLP fragments were then separated using gel electrophoresis.

TaqMan SNP Genotyping Assays (Applied Biosystems Inc, Foster City, CA, USA) were used to identify the genotypes of the IL-6 -572G/C and TNF-α -308A/G genes separately via the 7500 Fast Real-Time PCR System (Applied Biosystems Inc, Foster City, CA, USA). After PCR amplification, an endpoint plate reading using the Sequence Detection System Software (Applied Biosystems Inc, Foster City, CA, USA) was done for the measurements of fluorescence density, to plot the fluorescence (Rn) values based on the signals from each well. The plotted fluorescence signals indicate which alleles were in each sample.

### Genetic analysis

For the ACE gene, homozygote and heterozygote carriers of the ACE-D allele were grouped together and compared with patients without the ACE-D allele. Likewise for the ApoE gene, homozygote and heterozygote carriers of the ApoE ε4 allele were grouped together and compared with patients without any ApoE ε4 allele. For the IL-6 gene, carriers of the 572C allele (572CC and 572GC) were compared with patients of the 572GG genotype. Finally, for TNF-α gene, carriers of 308A allele (308AA and 308GA) were compared with patients of the 308GG genotype.

### Statistical analysis

The quantitative data were presented as mean±sd, while the qualitative data with frequency/percentage. Exploratory analyses were performed with independent t-test, Mann-Whitney test or chi-square tests, depending on the nature of data. A confirmatory analysis was performed with the generalized structural equation model (gSEM) [16], with demographics (age, gender, ethnicity), medical history (e.g., hypertension, diabetes, anaemia, renal disease), intraoperative factors (e.g., cardiopulmonary bypass time) and genetic factors included as covariates. The proposed model is essentially a network of equations, which could help to ascertain a covariate’s direct and indirect effects on the outcome. The final model was developed with a backward elimination procedure (removal probability ≥0.05). The cut-points of quantitative covariates (e.g., age, CPB time, eGFR) were identified based on the maximisation of the sensitivity and specificity. Analysis was done with IBM SPSS v.22.0 (IBM Corp, Illinois, USA) and Stata MP v.14 (Stata Corp, Texas, USA). All statistical analyses were conducted at 5% level of significance.

## Results

**Fig 1:**
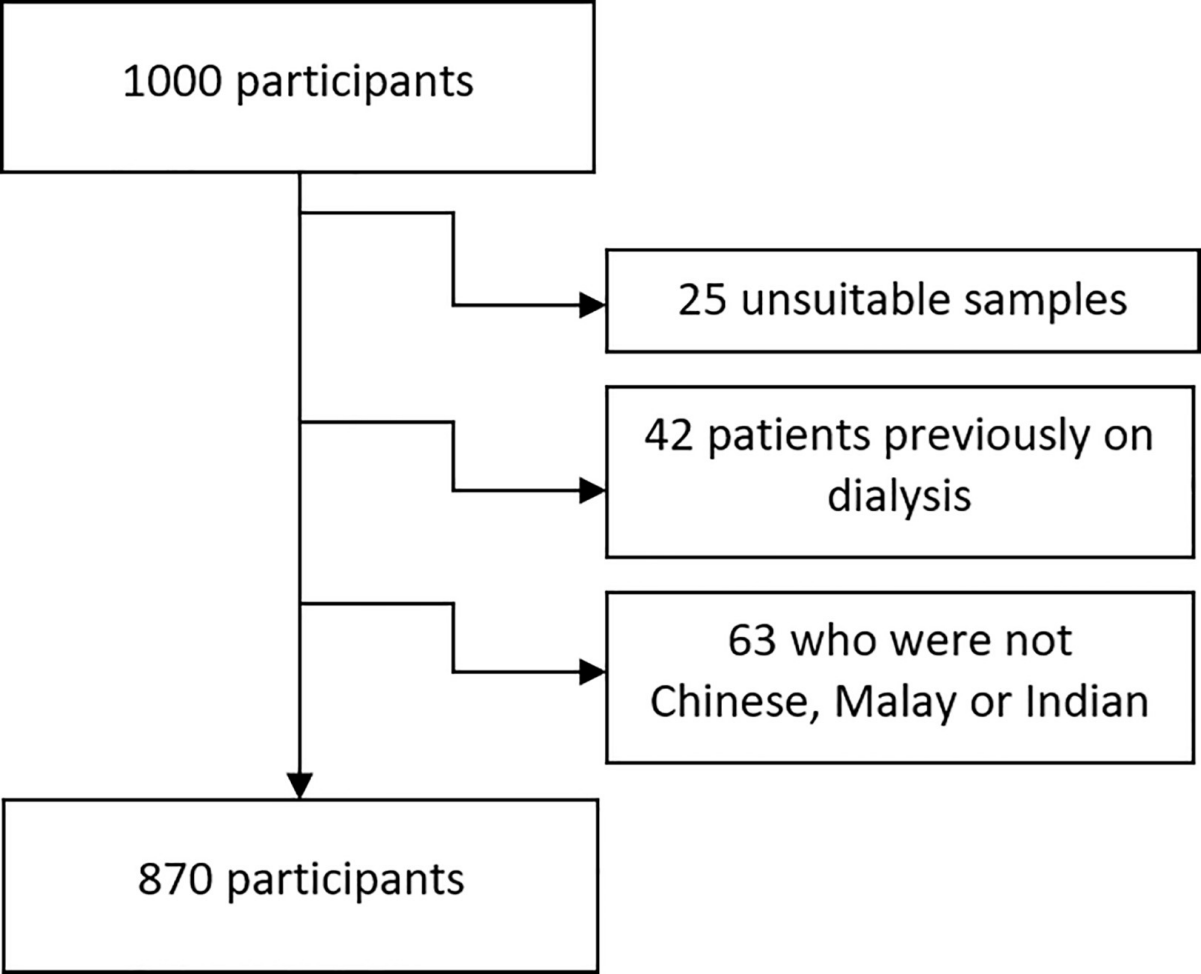
Flowchart on the final number of participants analyzed.

Between August 2008 and July 2010, a total of 1000 patients who underwent cardiac surgery consented for blood sampling for genetic analysis. However, 25 samples were technically unsuitable for analysis, and 105 patients were excluded as they were either on dialysis preoperatively (n = 42) or were not of Chinese, Malay or Indian ethnicity (n = 63). As such, the data of 870 patients were collated for analysis (Fig 1).

379 out of the 870 patients (43.6%) developed AKI–which was significantly associated with double the highest creatinine level in 7 days (183.2 vs 90.7 μmol/L, *p*<0.001) and more than 10-fold increased need for dialysis initiation (6.9% vs 0.6%, *p*<0.001) (Table 1).

**Table 1:** Study population characteristics.

	Overall (n = 870)	With AKI (n = 379)	Without AKI (n = 491)	*p*-value
Demographic variables				
Age (years)	59.33 ± 10.58	62.17 ± 9.86	57.14 ± 10.6	<0.001*
Race:				0.007*
Chinese	613 (70.46%)	246 (64.91%)	367 (74.75%)	-
Malay	156 (17.93%)	80 (21.11%)	76 (15.48%)	-
Indian	101 (11.61%)	53 (13.98%)	48 (9.78%)	-
Male gender	671 (77.13%)	289 (76.25%)	382 (77.8%)	0.59
Weight (kg)	66.15 ± 12.36	66.23 ± 13.19	66.1 ± 11.68	0.88
Height (cm)	162.64 ± 8.34	162.05 ± 8.55	163.1 ± 8.15	0.068
Body mass index (kg/m^2^)	24.96 ± 4.04	25.15 ± 4.3	24.81 ± 3.83	0.219
Preoperative co morbidities				
History of diabetes mellitus	405 (46.55%)	196 (51.72%)	209 (42.57%)	0.007*
History of hypertension	638 (73.33%)	316 (83.38%)	322 (65.58%)	<0.001*
History of renal impairment (eGFR < 90ml/min/1.73 m^2^)	559 (64.25%)	289 (76.25%)	270 (54.99%)	<0.001*
History of myocardial infarction	467 (53.68%)	232 (61.21%)	235 (47.86%)	<0.001*
History of congestive cardiac failure	177 (20.34%)	98 (25.86%)	79 (16.09%)	<0.001*
Preoperative LVEF (%)	50.09 ± 14.1	48.34 ± 14.37	51.44 ± 13.74	0.001*
Pre-operative haemoglobin (g/dL)	13.4 ± 1.78	12.85 ± 1.84	13.82 ± 1.61	<0.001*
Procedure				
CPB time (mins)	111.65 ± 50.09	123.29 ± 56.17	102.44 ± 42.55	<0.001*
Aortic cross clamp time (mins)	61.52 ± 36.71	67.74 ± 41.58	56.73 ± 31.68	<0.001*
Lowest haematocrit on CPB (%)	23.46 ± 5.44	22.67 ± 4.47	24.07 ± 6.02	<0.001*
Intra-operative RBC transfusion	283 (32.53%)	171 (45.12%)	112 (22.81%)	<0.001*
Usage of IABP	110 (12.64%)	69 (18.21%)	41 (8.35%)	<0.001*
Renal function variables				
Highest creatinine level in 7 days (μmol/L)	130.99 ± 79.97	183.22 ± 92.85	90.67 ± 30.83	<0.001*
New need for dialysis	29 (3.33%)	26 (6.86%)	3 (0.61%)	<0.001*
Genetic analysis				
Presence of ACE-D allele	516 (59.31%)	243 (64.12%)	273 (55.6%)	0.011*
Presence of IL6-572C allele	765 (87.93%)	317 (83.64%)	448 (91.24%)	0.001*
Presence of ApoE-ε4 allele	187 (21.49%)	85 (22.43%)	102 (20.77%)	0.556
Presence of TNF-α 308A allele	39 (4.48%)	32 (8.44%)	7 (1.43%)	<0.001*

eGFR–estimated glomerular filtration rate, LVEF–Left ventricle ejection fraction, CPB–Cardiopulmonary bypass, IABP–Intra-aortic balloon pump, RBC–Red blood cell.

* Statistically significant at 5%

As depicted in Table 1, patients with AKI were generally older (62.2 vs 57.1 years; *p*<0.001) and of non-Chinese ethnicity (35.1% vs 25.3%; *p* = 0.007), but there was no significant gender (*p* = 0.59) or anthropometric differences (*p* >0.05). Exploratory analyses showed that patients with AKI also had a higher prevalence of diabetes mellitus (51.7% vs 42.6%, *p* = 0.007), hypertension (83.4% vs 65.6%, *p*<0.001), renal impairment (76.3% vs 55.0%, *p*<0.001), myocardial infarction (61.2% vs 47.9%, *p*<0.001), congestive cardiac failure (25.9% vs 16.1%, *p*<0.001), lower preoperative left ventricle ejection fraction (48.3% vs 51.4%, *p* = 0.001), and a lower preoperative haemoglobin count (12.9 vs 13.8 g/dL, *p*<0.001). Intraoperatively, they had a longer CPB time (123.3 vs 102.4 min, *p*<0.001), longer aortic cross clamp time (67.7 vs 56.7 min, *p*<0.001), and a lower haematocrit during CPB (22.7% vs 24.1%; *p*<0.001). Patients with AKI were also associated with an increased likelihood of red blood cell transfusion (45.1% vs 22.8%, *p*<0.001) and initiation of intra-aortic balloon pump (IABP) (18.2% vs 8.4%, *p*<0.001). When analysed, presence of ACE-D allele (64.1% vs 55.6%, *p*<0.001) and TNF-α 308A allele (8.4% vs 1.4%, *p*<0.001) were associated with an increased risk of AKI. IL6-572C allele was found to be protective against AKI (83.6% vs 91.2%, *p*<0.001), while ApoE ε4 allele was not significantly associated with AKI (22.4% vs 20.8%, *p* = 0.556).

Confirmatory analysis found that old age (≥62 years), preoperative history of hypertension, anaemia (preoperative haemoglobin level of <12.5 g/dL) and renal impairment (<90 mL/min/1.73m^2^), and intraoperative prolonged CPB time (≥100 minutes) and use of IABP, were jointly and significantly associated with the development of AKI (Table 2). Other things being equal, patients with hypertension (adjusted odds ratio (AOR): 2.08), anaemia (AOR: 2.34) and renal impairment (AOR: 1.81) were twice likely to suffer from AKI. Prolonged CPB time (AOR: 1.85) and the use of IABP (AOR: 2.26) could also double the odds of developing AKI. In terms of genetic factors, ACE-D allele was associated with increased odds of AKI (AOR 1.59) while IL6-572C allele was significantly associated with lower odds of AKI (AOR: 0.57). ApoE ε4 and TNF-α 308A alleles were not significantly associated with AKI.

**Table 2:** Confirmatory analysis with generalized structural model (gSEM).

	Adjusted Odds Ratio	95% Confidence Interval	*p*-value
Age > 62 years	1.771	1.27–2.47	0.001*
Hypertension	2.083	1.46–2.988	<0.001*
Anaemia (Preop Hb <12.5g/dL)	2.338	1.67–3.28	<0.001*
Renal impairment (eGFR < 90 ml/min/1.73 m^2^)	1.807	1.28–2.56	0.001*
CPB Time >100 mins	1.848	1.35–2.52	<0.001*
IABP Used	2.256	1.41–3.61	0.001*
Presence of ACE-D allele	1.585	1.16–2.16	0.004*
Presence of IL6-572C allele	0.566	0.35–0.91	0.018*
Presence of ApoE-ε4 allele	1.036	0.71–1.51	0.853
Presence of TNF-α 308A allele	1.013	0.68–1.52	0.948

CPB–Cardiopulmonary Bypass, IABP–Intra-aorta balloon pump, eGFR–estimated glomerular filtration rate.

* Statistically significant at 5%

## Discussion

AKI after cardiac surgery occurs in a considerable proportion of the population undergoing cardiac surgery, with consequent significant morbidity and mortality. Increased age, history of hypertension, anaemia, renal impairment, longer CPB time and the use of IABP remained important risk factors for the development of AKI. Among the genetic polymorphisms, ACE-D allele was associated with an increased risk of AKI while IL6-572C allele was associated with a decreased risk of AKI.

The renin angiotensin system plays a crucial role in the regulation of renal blood flow and is pivotal in the ischemic injuries associated with cardiac surgery. Angiotensin II is a strong vasoconstrictor of the renal vasculature and its levels are influenced by circulating ACE levels, which is in turn, regulated by the ACE gene. Stafford-Smith et al had demonstrated an association of ACE-D allele to AKI among African Americans post cardiac surgery [10]. In a Turkish population, Isbir et al also demonstrated that ACE-D allele was associated with increased levels of ACE post cardiac surgery and consequently, AKI [13]. However, in a recent study of patients in the United Kingdom, Mcbride et al was unable to demonstrate any significant association of ACE-D allele to AKI [17]. We postulate that the study by Mcbride et al may have been underpowered to detect differences in associations with ACE-D alleles as there were only 48 samples for AKI compared to 330 samples for non AKI patients. Our study with 379 patients with AKI provides adequate sample sizes to reinforce the unfavourable role that ACE-D allele play in the overall development of AKI post cardiac surgery.

Following CPB, circulating endotoxins and cytokines peak between 4 to 24 hours. IL6 is widely regarded as one of the main proinflammatory cytokines influencing this inflammatory cascade. In particular, IL6-572C allele had been associated with increased circulating IL6 levels that result in an exaggerated inflammatory response. The ensuing accelerated atherosclerosis and direct damage to the kidneys leaves the patient at risk of AKI [10,18–20]. Of mention, Stafford-Smith et al [10] and Jouan et al [18], had both reported positive correlation of AKI with the presence of IL6-572C allele post cardiac surgery.

The importance of IL6-572C allele among the Asian population cannot be over stated–with up to 82.3% of Asians carrying the allele as compared to 5.0% among the Caucasian population [21]. In our study, 87.9% of our participants were found to be carriers of the IL6-572C allele, reaffirming the predominance of this allele in an Asian population. While IL6-572C had been positively associated with AKI in the Caucasian population (that had a low percentage of IL6-572C allele carriers), we had shown that IL6-572C conferred a divergent and protective role against the development of AKI, in our population (that had a high percentage of carriers).

Several studies had also identified this peculiarity on the role IL6-572C plays in the development of coronary artery disease (CAD)–a disease whose pathogenesis share several similarities to AKI. Hou et al, had in their meta-analysis, starkly pointed out that IL6-572C decreased the risk of CAD among the Chinese, but not among the Caucasians [22]. This phenomenon could well be related to a lowered IL6 level in patients with IL6-572C allele, (IL6-572CC and CG compared to those without, ie GG), as reported in a study among Korean men with CAD by Jang et al [23].

Drawing a parallel to the studies on CAD, we are certain that there exists modulating factors among Asians (including Southeast Asians), that had altered the phenotypic presentation of IL6-572C and its influence on AKI. This may be due to lowered IL6 levels and consequent dampening of the inflammatory response, decreasing the incidence of AKI. This unique protective role that IL6-572C exhibit possibly explains the high carrier rate we had in our Asian population. However, as our study is the first that had shown this association in AKI, further studies among the Asian and also Southeast Asian populations would be required to validate our findings.

ApoE polymorphisms had been hypothesised to play a role in modulating the inflammatory cascade in the development of renal injury. Non ApoE ε4 alleles in particular, have been linked to increased atheroma formation, while ApoE ε4 had been found to be protective against the development of AKI [24,25]. However, we did not demonstrate any association of ApoE to AKI in our population. This result mirrored the study conducted by Boehm et al, in a German cohort [26].

TNF-α 308A allele had also been widely associated with an increased inflammatory response and increased atherosclerotic phenomenon, directly inflating the risk of renal injury [27]. Bittar et al and Yoon et al both showed positive association of TNF-α –308A with AKI post cardiac surgery [28,29]. However, we did not demonstrate any association of TNF-α –308A with AKI in our population, also mirroring the results of Boehm et al [26].

Strengths of our study included being the first to have investigated the role of genetic polymorphisms and its association with AKI among a Southeast Asian population. Our local population is relatively homogenous and enjoys equal access to good medical care in our country, regardless of differences in socio-economic statuses. This allowed for adequate generalisability across our local population.

Some limitations of our study included the limited number of genetic polymorphisms that were tested. We had decided on the 4 genetic polymorphisms that had been widely studied and replicated at the time of genetic analyses. As our study focused on the association of the alleles on the development of AKI, we were unable to establish a definite causality of effect among the genes.

## Conclusion

We had demonstrated that the ACE-D allele was associated with an increased risk of AKI while the IL6-572C allele was associated with a reduced risk of AKI in a Southeast Asian population undergoing cardiac surgery. This contradictory effect of IL6-572C is a result of a complex interplay between the gene and population specific modulating factors. Our findings further underscored the necessity of taking into account population specific differences when developing prediction models for AKI.
